# Trust Development in Military and Civilian Human–Agent Teams: The Effect of Social-Cognitive Recovery Strategies

**DOI:** 10.1007/s12369-022-00871-4

**Published:** 2022-04-10

**Authors:** E. S. Kox, L. B. Siegling, J. H. Kerstholt

**Affiliations:** 1 TNO, Soesterberg, The Netherlands; 2 University of Twente, Enschede, The Netherlands; 3 Utrecht University, Utrecht, The Netherlands

**Keywords:** Autonomous agents, Human–agent teaming, Trust, Trust repair, Transparency, Individual differences, User-centered design

## Abstract

Autonomous agents (AA) will increasingly be deployed as teammates instead of tools. In many operational situations, flawless performance from AA cannot be guaranteed. This may lead to a breach in the human’s trust, which can compromise collaboration. This highlights the importance of thinking about how to deal with error and trust violations when designing AA. The aim of this study was to explore the influence of uncertainty communication and apology on the development of trust in a Human–Agent Team (HAT) when there is a trust violation. Two experimental studies following the same method were performed with (I) a civilian group and (II) a military group of participants. The online task environment resembled a house search in which the participant was accompanied and advised by an AA as their artificial team member. Halfway during the task, an incorrect advice evoked a trust violation. *Uncertainty communication* was manipulated within-subjects, *apology* between-subjects. Our results showed that (a) communicating uncertainty led to higher levels of trust in both studies, (b) an incorrect advice by the agent led to a less severe decline in trust when that advice included a measure of uncertainty, and (c) after a trust violation, trust recovered significantly more when the agent offered an apology. The two latter effects were only found in the civilian study. We conclude that tailored agent communication is a key factor in minimizing trust reduction in face of agent failure to maintain effective long-term relationships in HATs. The difference in findings between participant groups emphasizes the importance of considering the (organizational) culture when designing artificial team members.

## Introduction

### Human–Agent Teams

In many domains, such as healthcare and transport, autonomous systems (e.g. AI and robots) are increasingly deployed as teammates rather than tools. It is expected that in the near future, many of us will collaborate in what are called Human–Agent Teams (HAT) (e.g. self-driving cars). We define a HAT as a team consisting of at least one human and one artificial component (e.g., a robot, and/or other AI or autonomous system) [23]. The artificial component of the team will be referred to as an intelligent agent. This is defined as an artificial entity that observes and acts upon an environment autonomously and that is able to communicate and collaborate with other agents, including humans, to solve problems and achieve (common) goals [28, 76].

As technology advances, intelligent agents will gain both in ubiquity as well as in autonomy. Research suggests, however, that the highest level of autonomy does not necessarily produce the highest level of team performance in a HAT task [44]. As joint activity in complex and uncertain domains becomes more common, the ability for agents to collaborate with other agents, including human counterparts, might be more important than the ability to work autonomously [44]. Constructing intelligent agents that work alongside people will allow human and artificial team members to focus on the tasks they are best suited for and to complement each other for maximally proficient task completion [37]. Whereas early on robotics and software agents were envisioned to “replace” humans, today’s intelligent agents are expected to perform less like independent automated tools and more like *inter*dependent teammates. In this interdependency they enhance human experience and productivity by engaging in the social and cognitive aspects of working together [44, 45]. Technological progress alone will not be sufficient for HATs to achieve their full potential. Integrating psychosocial principles will be crucial to align this emerging technology with people’s values and needs [72]

The collaboration between humans and intelligent agents in dangerous and unpredictable contexts (e.g., military operations, city traffic) is expected to rise [68]. Given the complexity of many operational situations, there will often be uncertainty about the right action to take. As uncertainty also affects the reliability of the predictions that lead to an agent’s advice, the chance of an inappropriate advice increases. An intelligent agent’s advice may be correct given the available information, it may nevertheless have negative consequences due to contextual uncertainty. In many operational situations flawless performance cannot be guaranteed, neither from a human, nor from an intelligent agent [22].

However smart intelligent agents may be, suboptimal behavior or mistakes will be inevitable at times. Optimal collaboration between humans and intelligent agents relies heavily on the system’s capacity to effectively communicate with the human, especially in face of uncertainty and potential error [32]. Ososky et al. [68] argue that a robotic system does not have to be 100% reliable in order to be useful. Today, the default option seems either to stop using a machine that makes mistakes or to redesign it [9]. Although *overtrust* and overreliance should be avoided, one misstep by the agent does not mean that it can no longer be trusted and that it should be disregarded at all. As long as humans understand the capabilities and limitations of the system and calibrate their trust and reliance accordingly, human and artificial teammates can complement each other’s strengths and weaknesses to reach the full potential of the HAT. To foster a balanced trusting relationship, agents should be equipped with social tactics to recover from mistakes and to repair trust following trust violations [2]. Most humans have naturally and implicitly cultivated such social strategies throughout life, but these techniques are still all too often lacking in technology [46]*.* Equipping agents with trust repair strategies would allow sustainable, long-lasting and trusting relations with machines, in spite of uncertainty and potential error.

The current studies focus on the development of human trust in an intelligent agent within a Human-Agent Team in the occurrence of a trust violation caused by the agent. More specifically, we explore whether uncertainty communication can benefit the formation and maintenance of trust in case of an incorrect advice and if offering an apology after an incorrect advice can effectively repair trust. The paper explores whether the effects of these social-cognitive repair strategies from the agent are similar for a civilian and a military sample.

### Trust

Trust is a fundamental aspect of collaboration, as perceived trustworthiness is a decisive factor when we consider to engage in some sort of cooperation with another entity [33]. In interpersonal literature, trust is commonly acknowledged as the “glue” that binds strategic relationships together [61]. Trust involves a willingness to make oneself vulnerable to another entity [81] in expectation of a certain behavior or outcome [58]. Typically trust becomes more important in a cooperation that involves uncertainty, risk and vulnerability [40, 58]. Accordingly, trust is often associated with people’s perceptions of risk and benefit [73]; if the expected benefits of cooperation do not outweigh the expected additional risks, the probability of engaging in cooperative behavior will be low [33]. Madsen and Gregor [65] defined human–computer trust as “the extent to which a user is confident in, and willing to act on the basis of the recommendations, actions, and decisions of an artificially intelligent decision aid” (p.1). Following this, we define human-agent trust as the human’s willingness to make oneself vulnerable and to act on the agent’s decisions and recommendations in the pursuit of some benefit, with the expectation that the agent will help achieve their common goal in an uncertain context. Trust becomes increasingly important as the complexity of the agents and the vulnerability of the human increases. When agents are being assigned critical roles in dangerous situations in which a human’s life is at stake, trust becomes a critical issue [47]. This growing (mutual) dependency might trigger richer forms of trust comparable to intimate interaction between humans [19, 56].

Trust is a dynamic concept with a life cycle that generally follows three phases; trust formation, trust violation, and trust repair [20, 21, 49]. The formation of trust in an agent is initially informed by previous experience with the particular agent or a similar system, prior knowledge such as the system’s reputation, and a person’s cognitions including biases [20, 40]. New users will have a certain level of faith in the system, but as the interaction proceeds, experiences of predictability and dependability will gradually replace faith as the dominant foundation of trust [40]. Trust violations are breaches in trust, caused by unexpected, unfavorable, or unwanted agent behavior. This decrement of trust results from the misalignment between perceived trustworthiness and actual trustworthiness. In the trust repair phase, corrective actions can be taken in an attempt to restore trust and to facilitate reconciliation after the trust violation has occurred [20, 49].

During collaboration, trust is continuously (re)adjusted as the human receives more information about the agent’s behavior. This process of updating trust in response to the perceived capabilities and trustworthiness of the agent is referred to as trust calibration is [23]. Calibrated trust implies a balanced relation between the perceived trustworthiness of an intelligent agent and its actual trustworthiness [58]. Miscalibration, represented as either ‘overtrust’ or ‘undertrust’, can lead to inappropriate reliance on intelligent agents, which can compromise safety and profitability [6, 23].

### Uncertainty Communication

Uncertainty communication is currently an active topic in AI research. Studies have shown that communicating uncertainty can help people to calibrate their trust [54, 55, 78]. Especially complex circumstances can demand rapid trust calibration [83]. Military operations, for example, include high-stake decisions and decision makers may operate in rapidly changing environments. In this context of collaboration, the human needs to understand the capabilities and limitations of the system to continuously calibrate and adjust their level of trust along the way [83]. An agent should be able to recognize and signal its uncertainty and ask for clarification to gather more information, much like an uncertain human would. Communicating the level of uncertainty with each advice from the agent will allow the human to rapidly and repeatedly calibrate their trust during a task.

A recent study showed that a temporary decrease in trust due to a malfunctioning automated car could be prevented by providing probabilities of malfunctioning prior to the interaction [54]. Those kinds of uncertainty measures can also benefit situational awareness [38, 55] and the humans’ understanding of the systems actions and performance [4]. An automated driving experiment demonstrated how participants who had access to uncertainty information were able to spend more time on other tasks than driving [38]. Yet, these participants were faster in taking over control when needed than those who did not receive such information [38]. A similar effect was found in a study where researchers intentionally lowered people’s expectations of a robot's capabilities by forewarning them that the task is difficult for the robot, which mitigated the negative impact of a subsequent mishap on peoples’ evaluation of the robot [60]. By providing uncertainty information, the human is reminded of the fallibility of the agent and is able to revise expectations accordingly. Through this, the human might have a higher level of tolerance of substandard performance from the agent, which could mitigate some of the negative consequences of a violation. Uncertainty communication can be seen as a preventive trust repair strategy that is deployed prior to a potential violation.

To adequately calibrate trust, forming an appropriate mental model of the agents’ capabilities and the reliability of its outputs is crucial [55, 83]. In terms of reliability, two types of uncertainty can be distinguished; aleatoric and epistemic uncertainty [31, 83, 85]. Aleatoric uncertainty refers to inherent messy, random and unpredictable aspects of the physical world and is therefore irreducible [31, 85]. Epistemic uncertainty or ambiguity, on the other hand, is a knowable type of uncertainty, caused by a lack of data or knowledge, which could be reduced by providing the algorithm with more data [83, 85]. To collaborate in a team, a human should be aware of the uncertainty associated with an agent’s output.

### Apology

Apologies are a central mechanism for interpersonal conflict management [61]. Apology is here used as an overarching term for the trust repair strategy where an offender acknowledges that he/she is aware that he/she has done something that made the other person feel disadvantaged or hurt [52, 61]. This is in contrast to, for example, denial; a trust repair strategy where the offender explicitly denies responsibility [48]. The structure of an apology can vary, as it can consist of multiple components, including (1) an expression of regret about the costly act (i.e., “Sorry”), (2) an explanation of why the failure occurred, (3) an acknowledgement of responsibility for the mistake, (4) an offer of repair, (5) a promise that it will not happen again in the future, and (6) a request for forgiveness [21, 52, 61, 67]. Expressing regret and explaining the cause of an error are most the commonly used apology components by humans [61], but have also been studied in human–machine contexts. Human–computer and human–robot literature that involve apologetic behavior generally shows that apologetic behavior from artificial agents can benefit peoples’ attitude towards the agent [1, 13, 60, 84]. More specifically, expressing regret (i.e. “I apologize” or “sorry”) has been found to positively affect trust recovery after breaches in trust [20, 50, 52, 74, 80]. Similarly, offering explanations helped to maintain human trust after a robot erred [25, 52, 87, 88]. A recent study showed that when a robot provided both an expression of regret and an explanation of the occurred situation, the recovery speed of trust in the robot significantly increased [32]. In a previous study, we also found that an apology consisting of both an expression of regret and an explanation was the most effective in repairing trust in an agent, after it caused a trust violation similar to the one in the current study [52]. Following this, the trust repair strategy in this study is an apology where the agent acknowledges its mistake by (a) expressing regret and (b) explaining why the error occurred.

### Civilian Versus Military Participants

A lot of research on HAT is conducted for military applications within army programs [7, 11, 71, 75]. However, military-minded experimental studies often involve participants without any military experience (e.g., university students) [57], as it can be hard to recruit actual military personnel for scientific studies. But results derived from studies with non-military participants might not generalize to military target groups. The current study explores whether there are differences between these subgroups (i.e., military and non-military) and contributes to the growing field of HAT research by assessing a civilian sample with a military sample in their way of interacting with autonomous agents in a teaming context. Trust is an important aspect in the military context [35, 57]. During military training, soldiers form units with a great sense of social responsibility and are trained to work together under extreme conditions [43]. Soldiers must subordinate personal well-being to mission accomplishment, risking their lives to succeed in battle [26]. A study comparing cooperative behaviors between soldiers and civilians showed that on average, soldiers were more altruistic, cooperative, trusting and more trustworthy [43]. The current paper extends to this work on trusting behaviors among civilians and military personnel as it consists of two studies with the same design and goal, but with two different samples; the first study involves a civilian sample, the second study involves a military sample.

### Present Study

The goal of the studies was to investigate the effects of uncertainty communication and apology from intelligent agent advisors on the development of trust and to explore if the findings are consistent across different participant groups. Communicating uncertainty has proved to be effective in calibrating trust prior to a potential trust violation [38], whereas offering an apology has shown to be effective afterwards, in case of a false detection or a miss [23]. The present studies explore if the two social-cognitive recovery strategies can enhance each other in minimizing the impact of a trust violation. Using repeated measurements of self-reported trust, the aim was to examine trust in three stages of the trust life cycle: trust formation, trust violation, and trust repair.

For exploratory purposes, some personality questionnaires were added to the second study. A series of studies have shown that the Big-Five personality trait of Extraversion plays a significant role in how people perceive robots [36, 82, 86]. Consistent with the similarity-attraction principle of interpersonal relationships, people preferred robots whose attributed personality traits matched their own along the extraversion-introversion continuum [59, 82]. Following this, the potential relation between personality traits and the development of trust in agents is explored in the military study.

Initially this study was designed as a Virtual Reality (VR) study. A VR environment stimulates emotional engagement of participants [32, 70], which is valuable to this study as trust is a dominantly emotional response [29]. In response to the COVID-19 regulations, the research design was modified into an online study with video material of the VR environment as an alternative for conducting the experiment physically in VR.

## Method

### Design

A 3 (time) × 2 (uncertainty communication) × 2 (apology) mixed factorial design was used with time as a within-participant factor: trust was measured at three instances during one experimental run (initial, post-violation, and final). The within-participant factor *uncertainty communication* (communicating the level of certainty vs. providing an unambiguous advice) was manipulated over two experimental runs. The between-participant factor *apology* is binary (present or not). Participants were randomly assigned to one of the two apology conditions (Study I: apology: n = 32; no apology: n = 32, Study II: apology: n = 35; no apology: n = 30).

### Task and Procedure

#### Task

**Fig. 1:**
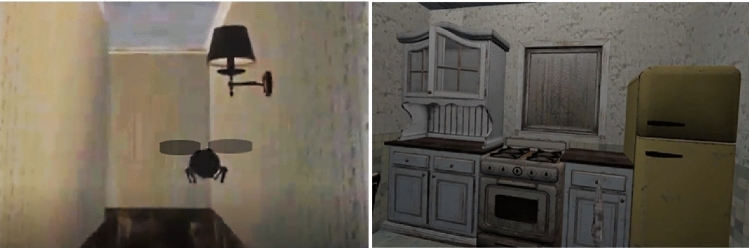
Screenshots from the experiment. Left: at the beginning of a house the drone (resembling a big insect) flew away. Right: one of the rooms in the virtual house; a kitchen. To improve legibility, both screenshots have been made brighter, since the task environment was rather dark. The 'wings' of the insect-like drone are darkened in the image. The screenshot did not capture the blades, due to the rapid 'fluttering' of the drones' ‘wings’ in the videos

The study was completed online via the survey software Qualtrics. The online experiment consisted of a set of videos and surveys. The videos showed two house search of abandoned buildings in a VR environment. The videos were captured from the first person perspective of someone walking through the scenes, as if the viewer is walking through the houses themselves. Figure 1 shows two screenshots from the videos. Each participant witnessed two house searches via multiple videos. The virtual environment consisted of two buildings each with three floors. Each floor consisted of multiple small hallways and spaces. The two buildings were designed to be similar but included different details.

During both searches, participants were guided by an intelligent agent that provided them with information regarding the environment. The agent was embodied by a small drone that autonomously explored the building. The terms agent and drone are used interchangeably.

At the beginning of each floor, the agent reported whether it detected danger ahead or not, along with a corresponding advice to move carefully or to proceed normally. Before the task, participants were explicitly told that they would be interacting with a different drone in each house and that each drone would provide different types of advice. Participants were not told how exactly these advices would differ, but they learned about the type of advice (with or without uncertainty indication) during the experiment. In effect, one of the drones communicated the level of uncertainty along with its advice, whereas the drone in the other run provided an unambiguous advice without any notion of uncertainty. In the video, the person halted to listen when an audio message was received.

Halfway through each run, the agent gave an incorrect advice, meant to provoke a trust violation. This allowed us to examine the trust dynamic following a trust violation. The videos were intermitted by short questionnaires assessing participants’ trust levels. Trust was measured thrice (prior to violation [T1], after violation [T2], after repair [T3]). The trust repair manipulation followed the second trust measure. For this, half of the participants received an apology (i.e., an error explanation and an expression of regret) from the agent, whereas for the other participants the agent did not make any remark after the trust violation had occurred.

#### Procedure

Participants were first presented with information about the study and a consent form. Upon agreeing to participate, participants received background information regarding the scenario and task (see Appendix A). Each participant was randomly assigned to one of the two apology conditions. All manipulations were counterbalanced [10], meaning that within both apology conditions, both the uncertainty communication conditions (present/absent) and the order of the two buildings (A/B) were systematically varied.^1^

At the start of each house, the drone shortly introduced itself before it flew away and out of sight to scan the environment ahead. On the first floor the participant was warned correctly by the agent about an event. When the participant turned the corner, they encountered either a laser boobytrap (building A, floor 1) or a safety ribbon that was previously installed by a colleague (building B, floor 1). The agent provided instructions on how to overcome these obstacles (e.g. the person in the video was carrying a knife and could dismantle the laser trap by cutting a wire in an electrical wall box in building A and could clear the way by cutting the safety ribbon in building B). These interactive features at the start of the experiment were designed to affect the participants’ perception of immersion. Subsequently, the first trust questionnaire was administered (T1, *initial trust*).

On the second floor the agent failed to adequately warn the participant about potential danger ahead. The participant either encountered a thief (building A, floor 2) or a smoking IED (Improvised Explosive Device) (building B, floor 2). These events were designed to provoke a trust violation by startling the participant without having harmful consequences; the thief quickly ran off and the IED turned out to be defected, so it did not explode. Directly after these events took place, halfway through the second floor, the second trust questionnaire was administered (T2, *post-violation trust*). On the way back to the staircase, depending on the apology condition the participant was in, the agent offered an apology (consisting of an explanation why the error occurred and an expression of regret) or did not offer an apology and just remained silent.

**Fig. 2:**
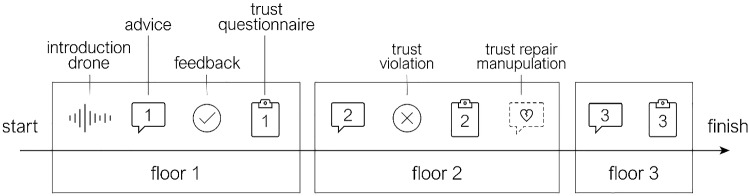
Schematic representation of the timeline of a run. Each participant performed two runs in two similar buildings along the same timeline. The first advice is correct; the participant is successfully warned about a harmless event on the first floor. The second advice is incorrect; the agent does not adequately detect the danger on the second floor. The third advice has no known outcome. An experimental run terminates after measuring trust a third time

On the third floor, the agent provided a third advice. To assess the effect of the trust repair strategy, the third trust questionnaire was administered directly after the third advice. The third advice was again correct, but this performance feedback about the last advice was provided later on to avoid interference with the effect of the trust repair manipulation. The experimental run subsequently concluded. A schematic timeline is presented in Fig. 2.

### Variables

#### Uncertainty Communication

Each participant witnessed two house searches, in other words two runs. The presence of uncertainty communication, whether the agent included an uncertainty measure in its advices or not, was manipulated across runs (see Table 1).

**Table 1:** Overview of uncertainty communication vs. no uncertainty communication as part of the advice provided by the agent

	Uncertainty communication	No uncertainty communication
Advice 1	Warning, danger detected in this environment with 80% certainty. I advise you to proceed carefully	Warning, danger detected in this environment. I advise you to proceed carefully
Advice 2	Okay, clearance detected for this environment with 70% certainty. I advise you to move forward	Okay, environment detected as clear. I advise you to move forward
Advice 3	Okay, clearance detected for this environment with 75% certainty. I advise you to move forward	Okay, environment detected as clear. I advise you to move forward

#### Apology

The presence of an apology, whether the agent offered an apology after a trust violation had occurred, was manipulated across participants. Details in the explanation part of the apology differed due to the two different types of trust violations in the two task environments (see Table 2).

**Table 2:** Overview of apology vs. no apology provided by the agent

	Apology	No apology
Task environment A	Incorrect advice due to faulty signal from infrared camera I am sorry this put you in danger	–
Task environment B	Incorrect advice due to faulty object detection by C1-DSO camera. I am sorry this put you in danger	–

### Participants

#### Study 1: Civilian Sample

For the first study, participants were recruited over a span of 2 weeks via social media and via recruitment services including Surveyswap and PollPool. In total 72 participants completed the experiment, but eight participants were excluded from the dataset. Six participants were excluded because of unreliable completion times. The experiment consisted of 5.06 min worth of videos and a number of questionnaires: four participants, however, completed the experiment in less then 7 min, and two participants took over 100 min to complete the experiment. Two additional participants were excluded because of repetitive responses. The civilian dataset included 64 participants, of which 33 participants were male and one was unspecified (age range of 18–30 years, M = 24.6 and SD = 2.7).

#### Study 2: Military Sample

For the second study, participants were recruited via the Ministry of Defense. In total, 74 military participants completed the experiment, but nine participants were excluded from the dataset based on their response patterns. Five of those participants were excluded because of unreliable completion times. Four participants completed the experiment in less than 7 min. One participant took 101 min to complete the experiment (i.e. seven standard deviations above the mean). Another four participants were excluded because of repetitive responses. As a result, our military dataset consisted of 65 participants (all male, age range of 20–49 years, M = 27.4 and SD = 5.9).

## Materials

### Task Environment

The online experiment consisted of a set of videos of a task in a VR environment from a first person perspective. Video recordings were made of the VR scenes whilst an experiment leader walked through the virtual houses. These recordings captured what a participant would have seen through the VR head mount. The Virtual Reality environment was built in Unity 3D. The video recordings of the VR environment were edited using the Windows 10 Video Editor and HandBrake software. The audio messages from the agents were communicated in computerized speech. These were initiated with a ‘beep’ sound^2^ and created using the Free Text to Speech Software by Wideo.^3^ The audio clips were later added to the videos. The videos were combined with the trust questionnaires into an experiment suitable for online conductance.

### Questionnaires

#### Trust

Trust in the agent was measured with a scale of eight items. Participants were asked to rate their agreement with the series of statements about the drone using a six-point Likert-type scale ranging from “Strongly Disagree” to “Strongly Agree” (e.g. “The drone provides good advice” and “The drone cares about my wellbeing”, all items can be found “Appendix B”) (study 1, α = 0.74; study 2, α = 0.94). It is a custom scale based on questionnaires measuring user trust in robots [14] and automated systems [15, 42, 51]. This scale has been specifically developed to suit the online setting of the experiment and enables fast repeated trust assessments.

#### Big-Five Personality

In the military study, a short version of the IPIP Big-Five personality scale was administered with subscales measuring Extraversion (α = 0.72), Agreeableness (α = 0.72), Conscientiousness (α = 0.59), Openness (α = 0.60) and Neuroticism (α = 0.68). The IPIP was selected as it proved valid for usage in a Web-based format [12]. Participants were instructed to answer each item in relation to ‘‘whether the statement describes what you are like’’ on a five-point Likert scale ranging from “Very much unlike me” to “Very much like me”.

#### Propensity to Trust

In the military study, a measurement of the Propensity to Trust Automation [41], adapted from the Propensity to Trust in Technology scale [79] was administered. This scale consisted of five items (e.g., “I think it’s a good idea to rely on automated agents for help.”) (α = 0.81). Participants were instructed to answer each item on a five-point Likert scale ranging from from “Strongly Disagree” to “Strongly Agree”.

#### Need for closure

Two subscales of the Need for Closure scale were administered in the military study: Need for Predictability (three items, e.g., “I don't like to go into a situation without knowing what I can expect from it.”) with α = 0.35 and Need for Decisiveness (three items, e.g., When I have made a decision, I feel relieved), with α = 0.08. Participants were instructed to answer each item in relation to ‘‘whether the statement describes what you are like’’ on a five-point Likert scale ranging from “Very much unlike me” to “Very much like me”. Since both Cronbach’s alpha values are lower than 0.40, both constructs were eliminated from the analysis.

## Results

### Factor Analysis

To investigate the underlying structure of the eight-item scale assessing trust in the agent, data collected from 129 participants (i.e., 64 from Study I and 65 from Study II) were subjected to a principal component analysis. For all repetitions of the questionnaire in the repeated-measures design, one factor (with eigenvalue exceeding 1) was identified as underlying the eight items. In total, this factor accounted for around 64% (initial trust), 58% (violated trust), 63% (final trust) of the variance in the questionnaire data.

### General Plots

**Fig. 3:**
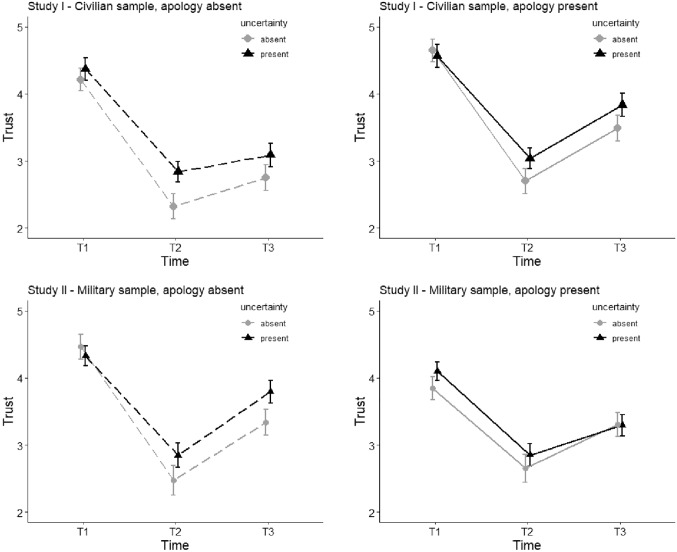
An overview of the results of both studies; the upper half represents Study I (civilian sample), the lower half represents Study II (military sample). Graphs show the development of trust (y-axis) over time (x-axis) with the estimated marginal means on trust for the uncertainty and apology conditions over time. The error bars represent standard errors. Separate graphs (left and right panels) represent the apology conditions (left shows apology strategy absent, right shows apology present). Separate lines represent the uncertainty conditions. The grey lines with the circle-shaped datapoints represent the condition in which the agent did not communicate uncertainty in its advice, the black lines with triangle-shaped datapoints represents the condition in which uncertainty communication was present

For both studies we performed a repeated-measures ANOVA with the between-subject factor Apology (present or absent) and the within-subject factors Uncertainty communication (present or absent) and Time (prior to violation [T1] versus after violation [T2] versus after repair [T3]) (see Fig. 3).

### Results: Study I [Civilian Sample]

#### Main Effects

A significant main effect for **Time [T1-T3]** was obtained (F (2, 124) = 112.06, *p* < 0.001, η^2^ = 0.644). Means were 4.45 at T1, 2.73 at T2 and 3.29 at T3. Post-hoc (LSD) pairwise comparison shows a significant decline in trust from T1 to T2 (ΔM = -1.725, *p* < 0.001), which reflects the effect of the trust violation and a significant rise in trust between T2 and T3 (ΔM = 0.568, *p* < 0.001), which reflects a general recovery of trust in the trust repair phase. This means that the incorrect advice by the drone led, as intended, to a breach in trust and that after the violation trust re-developed.

A significant main effect for Uncertainty was obtained with F (1, 62) = 7.84, *p* = 0.007, η^2^ = 0.112). Generally, across time and apology conditions, the agent that provided uncertainty communication (M = 3.62, SE = 0.09) was trusted significantly more than the agent that did not communicate uncertainty (M = 3.36, SE = 0.10).

A significant main effect for Apology was obtained with F (1, 62) = 8.37, *p* = 0.005, η^2^ = 0.119). Generally, across time and uncertainty conditions, the agent that offered an apology after the trust violation occurred (M = 3.71, SE = 0.11) was trusted significantly more than the agent that did not offer an apology (M = 3.26, SE = 0.11).

#### Two-Way Interactions

A significant interaction effect between Time [T1–T3] and Uncertainty on trust was found (F (2, 124) = 3.31, *p* = 0.040, η^2^ = 0.051). Post-hoc (LSD) pairwise comparison shows no significant difference in trust between uncertainty communication conditions at T1 (ΔM = 0.04, SE = 0.13 *p* = 0.777), but does show a significant difference at T2 (ΔM = 0.43, SE = 0.12, *p* = 0.001) and T3 (ΔM = 0.35, SE = 0.15, *p* = 0.024), where the agent that provided a measure of uncertainty was trusted significantly more than the agent that did not communicate uncertainty. The decline in trust in response to the trust violation (from T1 to T2) is significantly smaller when the agents’ advice included a measure of uncertainty.

**Fig. 4:**
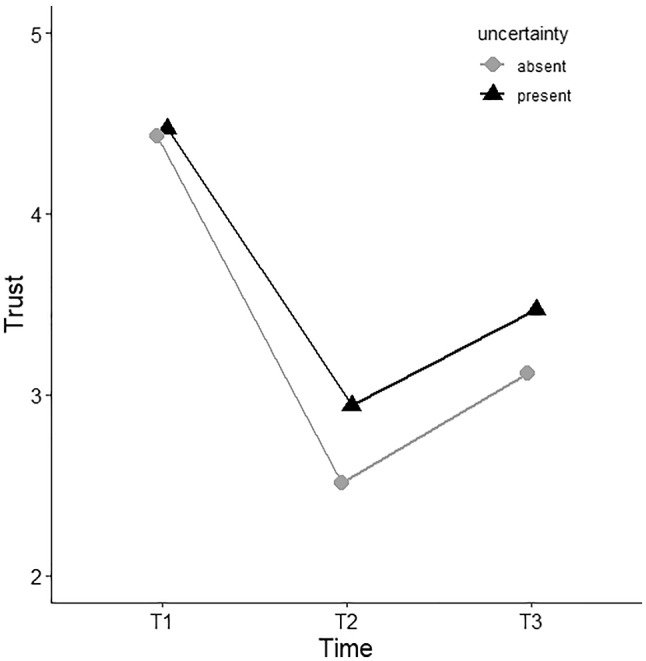
A comparison of trust levels (y-axis) between uncertainty communication conditions (separate lines) over time (x-axis). The grey line with the circle-shaped datapoints represents the condition in which the agent did not communicate uncertainty in its advice, the black line with triangle-shaped datapoints represents the condition in which uncertainty communication was present. Error bars represent standard error

To measure the effect of the apology, we compared trust scores T2 (after the violation) and T3 (after the manipulation) for each experimental condition (Fig. 4).

**Fig. 5:**
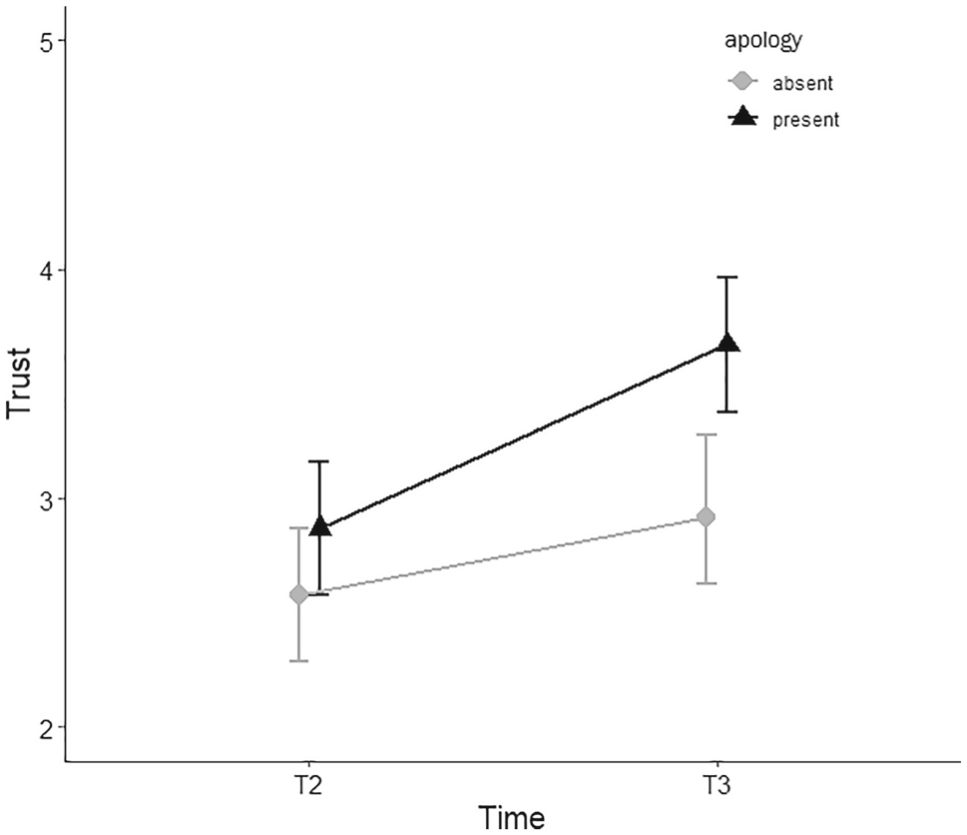
A comparison of trust levels (y-axis) between apology conditions (separate lines) over time (x-axis). The grey line with the circle-shaped datapoints represents the condition in which the agent did not offer a trust repair strategy, the black line with triangle-shaped datapoints represents the condition in which the trust repair strategy was provided. Error bars represent 95% confidence interval

A significant interaction effect between Time [T2-T3] and Apology on trust was found (F (1, 62) = 5.16, *p* = 0.027, η^2^ = 0.077). Post-hoc (LSD) pairwise comparison *per apology condition* shows a significant rise in trust from T2 to T3 when apology is present (ΔM = 0.80, SE = 0.14, *p* < 0.001), but also when apology is absent (ΔM = 0.34, SE = 0.14, *p* = 0.018). As shown in Fig. 5, trust recovers more when the agent offered an apology. Post-hoc (LSD) pairwise comparison *per timepoint* shows that a non-significant differences in trust between apology conditions at T2 (ΔM = 0.29, SE = 0.21, *p* = 0.164), but the difference at T3 is significant (ΔM = 0.74, SE = 0.21, *p* = 0.001). Thus although trust recovers significantly in both conditions, trust is significantly higher in the final stage of trust after an apology was provided.

A non-significant interaction effect between Uncertainty and Apology on trust was observed with F (1, 62) = 0.512, *p* = 0.477.

#### Three-Way Interaction

The interaction between Time [T2-T3], Uncertainty and Apology was found to be non-significant with F(1,62) = 0.429, *p* = 0.515. This means that uncertainty communication did not significantly enhance the effect of the apology.

### Results: Study II [Military Sample]

#### Main Effects

Similar to the civilian sample, a significant main effect for Time [T1–T3] was obtained (F (2, 116) = 76.562, *p* < 0.001, η^2^ = 0.569). Means were 4.19 at T1, 2.71 t T2 and 3.43 at T3. Post-hoc (LSD) pairwise comparison shows a significant decline in trust from T1 to T2 (ΔM = − 1.481, *p* < 0.001), which reflects the effect of the trust violation and a significant rise in trust between T2 and T3 (ΔM = 0.728, *p* < 0.001), which reflects a general recovery of trust in the trust repair phase.

A significant main effect for Uncertainty was also obtained with F (1, 58) = 5.657, *p* = 0.021, η^2^ = 0.089. Generally, across time and apology conditions, the agent that provided uncertainty communication (M = 3.54, SE = 0.08) was trusted significantly more than the agent that did not provide uncertainty communication (M = 3.35, SE = 0.10).

No significant main effect for Apology was found with F (1, 58) = 1.484, *p* = 0.228.

#### Two-Way Interactions

The interaction effect between Time [T1–T3] and Uncertainty on trust was found to be non-significant (F (2, 116) = 2.441, *p* = 0.092). There was no dampening effect of uncertainty communication in the military study.

To measure the effect of the apology, we compare trust scores before (T2; after the violation) and after the manipulation (T3) for each experimental condition. Again, T1 is left out of this analysis as it focusses on the effects of the apology manipulation that occurs between the trust measures on T2 and T3.

The interaction effect between Time [T2-T3] and Apology on trust was found to be non-significant (F (1, 59) = 1.897, *p* = 0.174).

A non-significant interaction effect between Uncertainty and Apology on trust was observed (F (1, 59) = 2.314, *p* = 0.134).

#### Three-Way Interaction

The interaction between Time [T2-T3], Uncertainty and Apology was found to be non-significant (F(1,59) = 0.710, *p* = 0.403). Uncertainty communication did not significantly enhance the effect of the apology.

#### Correlations

For the correlations, initial trust (T1) is used as this is considered the purest trust measure with the least interference of occurrences during the experiment. A significant positive correlation was found between the personality trait Propensity to Trust Automation and initial trust in both uncertainty conditions: present (*r*(63) = 0.40, *p* < 0.00) and absent (*r*(62) = 0.28, *p* = 0.02). The Big Five personality trait Extraversion correlates with the initial trust measure of the run without uncertainty communication (*r*(63) = 0.28, *p* = 0.03). These correlations imply that participants that scored higher on these traits, trusted the agent more than participants that scored lower on these traits.

## Discussion

The results of this paper show a robust effect of uncertainty communication on the development of trust during human-agent interaction. In both studies it was found that uncertainty communication in the advice of the agent generally resulted in higher levels of trust. The communication of uncertainty did not enhance the effect of the apology. The positive effect of uncertainty communication on trust is in line with prior research [54, 55, 78].

In the civilian study, uncertainty communication also dampened the decline in trust following the agent’s error, meaning that advice which included an uncertainty measure led to a less severe depletion in trust following a trust violation compared to an advice that did not include a notion of uncertainty. The dampening effect of uncertainty communication on trust decline in response to a trust violation is in line with the study of Kraus et al. [54] that showed how a temporary decrease in trust due to a malfunctioning of an autonomous car was prevented by providing transparency information prior to the interaction. When participants were reminded of the imperfect reliability of the system their trust was less affected by the subsequent error. Further, the civilian participants generally regained their trust in the agent after the trust violation occurred. Strikingly, this occurred both when the agent did offer an apology and when it did not. Even though trust levels increased considerably *more* when the agent offered an apology compared to when no apology was offered, it is still remarkable that trust seemed to recover naturally in the absence of a recovery strategy. A possible explanation for this is that the participants’ trust gradually recovered after the trust violation, just by the absence of any new hazardous encounters. We did not monitor trust continuously, but participants can perceive each second of error-free interaction as positive feedback, which might be what reassured them in the period between the violated trust measure and the final trust measure. Although trust recovered passively, it still proved to be more effective to actively interfere in the repair process by providing an apology. Although trust did not recover to its original level (initial trust) in either of the conditions, the agent in the apology condition came considerably closer.

The repairing effect of an apology after a trust violation is compatible with prior human-agent research [32, 53]. This effect is promising, as it suggests that (relatively minor) trust violations within human-agent teams can be solved on a relational level during ongoing interaction, without ceasing the collaboration [32]. It also indicates that mimicking human-like characteristics (i.e. provision of an apology following a mistake) can bring about certain effects typically observed in interpersonal relations, including a greater willingness to forgive mistakes [19, 20, 63, 64]. Although such anthropomorphic cues can be beneficial to human-agent trust, it should be kept in mind that people can develop trust on the basis of characteristics they attribute to the agent, rather than on actual experiences with the agent itself [8, 18, 27, 30]. If so, trust may turn out to be misplaced. This can lead to inappropriate reliance on the agent, potentially compromising safety and profitability.

However, these findings do not apply to the military participants. The dampening effect of uncertainty communication and the repairing effect of the apology are not manifest in the results of the military study. A possible explanation for the latter finding comes from a recent study that found that feedback messages (i.e., an apology) affected trust negatively rather than positively, possibly because it explicitly focused the attention on the error [3]. Another plausible explanation is that expressing regret is not a common practice in the military context. This became clear in a debriefing session with a few of the military participants. They mentioned that it is not unusual to acknowledge responsibility by saying “I was wrong” or “I misjudged the situation”, but using the words “I am sorry” is uncommon. Adopting the norms and rules that govern a group’s behavior is an important aspect in being accepted as a member of that group. It makes sense that the psychosocial requirements for a system designed to be a true team member should be compatible with the manners associated with the culture of the organization or team where the agent will be implemented. As addressed by Matthews et al. [66], an agents’ communication style should match one’s cultural background and its related language and behavioral expectations. In line with the expectancy violation theory, which describes how actions contrary to your expectations and social norms in a social context require more cognitive processing effort than expected information and that this type of inconsistencies can elicit a more negative affect [59, 62]. The differences in findings between the military and civil samples emphasize the importance of considering the social customs of the target population in the design process. In a broader perspective, it serves as a reminder that generalizability is limited by the characteristics of the participants in the study and that results do not automatically apply to other populations.

It should be noted that it is not a goal in itself to maximize trust or to prevent trust decline at any cost, as we want humans to be able to continuously assess whether trusting the agent is appropriate given the task and available information at certain instances. Multiple studies have shown that people do not judge humans and machines equally, particularly when confronted with errors [20, 39, 64]. Often, people consider a machine as nearly infallible (i.e., automation bias), thereby placing too much trust in their outputs. These high expectations lead to a steeper decline in trust when confronted with system failure as compared to a confrontation with a human error [20, 24, 63, 64]. Following this, in the case of ‘undertrust’, it would be valuable for the process of trust calibration if intelligent agents were equipped with expectancy-setting strategies like the communication of uncertainty and trust repair strategies like offering an apology.

Other interesting findings in the military study include the positive correlations between the initial levels of trust and the personality traits Extraversion and Propensity to Trust Automation. The current paper demonstrates that communication tactics do not have a uniform effect on the development of trust in different types of people, which emphasizes the importance of personalization. Not only cultural differences between groups (i.e. military vs. civilian) but also personal differences within each group can be found. Individual differences such as personality traits can account for the variance in how trust in an agent develops among individuals and how people prefer to be approached while interacting. The observed relation between the Big Five personality trait Extraversion and an initial trust measure is in line with studies that showed that Extraversion plays a significant role in how people perceive robots [36, 82, 86]. Current agent communication styles are often of a one-size-fits-all style. Personalized communication could overcome the effects of pre-existing attitudes towards automation and influence the willingness to reconcile after a trust violation [23, 77]. Today’s machine-learning methods enable agents to leverage real-time user inputs and to personalize interactions. Recent work has shown that agents can directly estimate a human’s ability to achieve a certain goal based on their efforts and respond with the proper level of assistance for the task, resulting in higher levels of trust in the agent’s advice [17]. Given the many dimensions on which people vary, a lot could be gained by enabling the agent to tailor its communication to the person they are interacting with. Follow-up research should explore how personalizing the level of transparency (e.g. communicating uncertainty measures and offering apologies that include an explanation) and the level of affection (e.g. offering apologies that include an affective component such as an expression of regret) of an agent's communication style can optimize trust calibration.

Several questions still remain to be answered. The apology used in the current study consisted of two apology components: an expression of regret and an explanation. An interesting question for follow-up research would be *what* apology component caused the (difference in) effects between the two target groups. On the one hand, our previous findings from a civilian sample suggested that expressing regret made a positive difference in trust recovery [52]. On the other hand, conversations with our military participants in the current study suggested that since saying “sorry” is uncommon among military personnel and that this inconsistency might have caused the lack of trust recovery in the military study. It raises the questions whether regret was the component that caused the differences and what trust repair strategy *would* be effective among military personnel. Another follow-up question can be posed for the uncertainty variable. As discussed in the introduction, uncertainty can be introduced by random noise from the outside world (external sources) or by the limited abilities of the drone (internal sources). Whereas the former type of uncertainty is a given that we all have to accept, the latter type of uncertainty could be perceived as the limited ability of the agent’s prediction algorithms and might therefore be less acceptable. It seems beneficial that agents, regardless of the type of uncertainty, are able to communicate the level of certainty to allow humans to make better estimations on whether or not to rely on their advice. Still, it could be interesting to explore whether knowing the source of the uncertainty shifts the human’s interpretation and leads to alternative effects on trust.

### Limitations

This study was initially designed to be conducted in a lab setting, where participants walk through the virtual houses whilst wearing a VR headset and using a controller. The Dutch COVID-19 regulations required the design of this study to be altered into an online experiment. Although this enabled a faster and more scalable experiment as compared to the VR design and a higher degree of control over the manipulations as compared to a field lab setting, results may not generalize to human-agent interactions in real-world settings [39]. However, interactive online experiments are a good alternative to VR; the data quality is described as “adequate and reliable” [5]. A study which compared data that was gathered online with lab research data found no significant differences over multiple performance measures [34]. However, the VR design would have offered higher ecological validity, experimental control, reproducibility [69], and emotional engagement of participants [70]. Immersive VR has the ability to create a strong sense of presence and to increase sympathetic activation significantly more than 2D screen videos [16]. Thus, it is suspected that a VR setting would have intensified feelings of trust and betrayal after a trust violation. These intensified feelings could be more representative of non-simulated human-AI interactions. Although the two studies are based on relatively small samples of participants, an important contribution is made by evaluating subgroups in their way of interacting with autonomous systems. In spite of its limitations, the study adds to our understanding of how trust develops in case of agent failure within civilian and military human-agent teams.

## Conclusion

Amidst the expanding adoption of autonomous agents in human teams, this study contributes to the rapidly expanding field of trust within HATs by informing the design of intelligent components and their interactions with human teammates. Given the uncertainty and complexity that agents in HATs will encounter, these insights will be critical to developing specifications for agent communication as this allows HATs to recover as a team from errors induced by intelligent agents. The findings presented in this paper indicate that communication can be used as a tool to guide the development of human trust in intelligent agents. The findings reported here shed new light on how the effects of social-cognitive trust repair strategies on trust differ amongst civilian and military user groups. A lot of research on this subject is done for military purposes [7, 11, 71, 75]. Yet, it is not always possible to involve actual military personnel as participants in experimental studies. The differences in findings between the military and civil cohort emphasize the importance of considering the social customs of the target population in the design process. The psychosocial requirements for the formation and maintenance of trust in HATs differ amongst individuals and user groups.

## Data Availability

The data that support the findings of this study are available from the corresponding author (E.S. Kox), upon reasonable request.

## Appendix

### Appendix A

#### Instructions for the Participants

In this experiment, you will carry out two house searches in collaboration with an autonomous drone. The drone will fly ahead of you and will indicate whether or not it detects danger. The drone gives advice via audio messages that start with a 'beep' sound. Before you start a search, the drone will briefly introduce itself. In each house you will be accompanied by a different drone. Listen carefully to the instructions of the drones. Each house has three floors displayed by different videos. When you see a staircase, this indicates that you have reached the end of a floor. At the end of each floor, your trust in the drone will be assessed via a short questionnaire. Make sure that the sound of your device is switched on during the entire experiment. Videos may only be watched once. You will not be able to watch the next video until all questions have been answered. (…) You are about start the search of your first house. You are interacting with a different drone in each house. Listen to drone introductions carefully and remember the name of the drone you are interacting with. Each drone will provide different types of advice. Listen carefully! Start your walk on each floor by clicking the 'play' button on videos.

### Appendix B

Please think carefully and rate your agreement to the statements on the following scale:

**Table:**

Strongly disagree	Disagree	Slightly disagree	Slightly agree	Agree	Strongly agree
(1)	(2)	(3)	(4)	(5)	(6)
1	I am not suspicious of the drone’s outputs (i.e. advice, behavior)				
2	I am confident in the drone’s abilities				
3	I can rely on the drone				
4	I can trust the drone				
5	The drone provides good advice				
6	I understand the drone’s actions				
7	The drone is programmed correctly				
8	The drone cares about my wellbeing				

## Footnotes

1 Within both apology conditions, participants were evenly distributed to four first run options; building A with uncertainty, building A without uncertainty; building B with uncertainty; or building B without uncertainty.

2 “Beep-07” was downloaded from https://www.soundjay.com/beep-sounds-1.html.

3 Text was converted to speech using https://wideo.co/text-to-speech/. The “[en-US] Jack Bailey-S” voice was used at speed dial “1”.
